# Harnessing Dengue Rapid Diagnostic Tests for the Combined Surveillance of Dengue, Zika, and Chikungunya Viruses in Laos

**DOI:** 10.4269/ajtmh.19-0881

**Published:** 2020-03-09

**Authors:** Manivanh Vongsouvath, Tehmina Bharucha, Malee Seephonelee, Xavier de Lamballerie, Paul N. Newton, Audrey Dubot-Pérès

**Affiliations:** 1 Lao-Oxford-Mahosot Hospital-Wellcome Trust Research Unit (LOMWRU), Microbiology Laboratory, Mahosot Hospital, Vientiane, Lao PDR;; 2 Institute of Glycobiology, Department of Biochemistry, University of Oxford, Oxford, United Kingdom;; 3 Unité des Virus Émergents (UVE: Aix-Marseille Univ-IRD 190-Inserm 1207-IHU Méditerranée Infection), Marseille, France;; 4 Centre for Tropical Medicine and Global Health, Nuffield Department of Medicine, University of Oxford, Churchill Hospital, Oxford, United Kingdom

## Abstract

Recent expansions of vector-borne diseases highlight the need for improved surveillance, especially in resource-poor settings. Dengue virus (DENV), chikungunya virus (CHIKV), and Zika virus (ZIKV) share the same vectors as well as similar clinical presentations, suggesting that combined surveillance would be useful. We hypothesized that blood spotted on dengue rapid diagnostic tests (RDTs) could be harnessed for sample collection in remote areas for subsequent detection of DENV, CHIKV, and ZIKV by reverse transcription real-time polymerase chain reaction (RT-qPCR). CHIKV and ZIKV dilutions were spotted on dengue RDTs (SD BIOLINE Dengue DUO, Standard Diagnostics, Gyeonggi-do, Republic of Korea), dried, and extracted. As reference, aliquots of each viral dilution were directly extracted. Using specific RT-qPCR tests, both viruses were successfully detected from RDT extracts. However, the limit of detection was slightly lower in comparison to direct extracts, two logfold for CHIKV and one logfold for ZIKV. For analysis of temperature stability, DENV dilutions were spotted on RDTs and stored for up to 2 months at −80°C, 4°C, or 35°C before testing. Storage of RDTs for 2 months at 35°C did not compromise detection of RNA by RT-qPCR; only minimal degradation was observed. This proof-of-principle study demonstrates the potential of using dengue RDTs for DENV/CHIKV/ZIKV combined surveillance in areas without access to laboratory facilities. Further investigations are needed for evaluation of tri-viral surveillance under field conditions using patient samples. Large-scale implementation of surveillance for these viruses is of crucial public health importance for the early detection of epidemics. This method also has important implications for improving understanding of the molecular epidemiology of the three viruses.

## INTRODUCTION

Dengue virus (DENV) infection is hyperendemic in tropical and subtropical areas, with an estimated 390 million patients per year.^1,2^ There is recent evidence to suggest alarming global expansion of two other mosquito-borne viruses, chikungunya (CHIKV) and Zika (ZIKV) viruses.^3–5^ In Southeast Asia (SEA), urban outbreaks of CHIKV have been well documented in the past decade, but epidemiology in rural areas is not well characterized.^6^ Zika virus is considered to be endemic at low prevalence in SEA. With only limited data available, ZIKV circulation is probably underestimated.^7^ However, a few countries reported increasing number of patients in 2016–2017, 686 in Thailand, 493 in Singapore, 232 in Vietnam, and 57 patients in the Philippines.^8^ The three arboviruses are transmitted by the same mosquito vectors, the *Aedes* species, and consequently have overlapping geographical distributions.^9^ They also share remarkably similar clinical presentations, with common symptoms reported as fever, headache, vomiting, rash, myalgia, and severe arthralgia. For these reasons, strategic public health programs advocate for combined surveillance and control efforts.^9^

Reverse transcription real-time polymerase chain reaction (RT-qPCR) assays are the mainstay for specific detection of DENV, CHIKV, and ZIKV acute infection. However, in many resource-limited settings of high disease burden, there is no access to laboratory facilities, especially in rural areas. Point-of-care testing with antibody (Ab)/antigen (Ag)-detecting rapid diagnostic tests (RDTs) is increasingly available and has demonstrated considerable potential for surveillance of diseases such as DENV, but not yet for CHIKV or ZIKV.^10,11^

We aimed to produce a pragmatic and low-cost approach to the combined surveillance of the three viruses in rural areas, not only suitable for the Lao PDR (Laos) but also applicable to other resource-limited settings. Successful DENV RNA detection by RT-qPCR from positive dry dengue RDTs used for routine diagnosis in a provincial hospital has previously been described by our group.^12^ Here, we aimed to evaluate RT-qPCR detection of both CHIKV and ZIKV from dengue RDTs and to assess the temperature stability of the detection of DENV in RDTs by RT-qPCR. We used serial viral dilutions that were spotted onto dengue RDTs. RNA detection using specific RT-qPCR after extraction from RDTs was compared with direct extractions for all virus dilutions. Rapid diagnostic tests loaded with serial dilutions of DENV solutions were stored at different temperatures, 4°C, −80°C, and 35°C, for up to 2 months before testing by RT-qPCR.

## MATERIALS AND METHODS

### Description of RDT.

The SD Dengue DUO RDT (Standard Diagnostics, Kyonggi-do, Korea) is an in vitro immunochromatographic assay for the detection of DENV NS1 Ag and anti-dengue IgM/IgG antibodies in human serum, plasma, or whole blood, from finger-prick or venous blood. This test comprises a pair of test devices, a DENV NS1 Ag test on the left side, and a DENV IgM/IgG Ab test on the right side. Each device contains a strip, enclosed in a plastic cassette. The strip is made of three compartments: 1) an absorptive pad where the patient sample (serum, blood, or plasma) is applied and which then moves along the strip; 2) a conjugate or reagent pad which contains antibodies, specific to the targeted analyte, conjugated to colored particles; and 3) a nitrocellulose membrane on which the immunocomplexes move until the zone of reaction where they are immobilized and appear as a colored band. The test is easy to perform—three drops (using a dropper provided with the kit, ∼100 μL) and 10 μL (using a capillary provided with the kit) of the sample are applied into the two small wells on the NS1 and Ab cassettes, respectively. Four drops of the diluent (provided with the kit) are then applied on the Ab cassette. The test results are obtained in 15 minutes.

### Virus dilutions and spotting on RDT.

DENV serotype 1 isolated in cell culture from a dengue patient, as previously described,^12^ was 10-fold serial-diluted using Minimum Essential Media (MEM, Gibco, Thermo Fisher Scientific, Waltham, MA). Three consecutive dilutions (“high titer” = H, “medium titer” = M, and “low titer” = L) were selected based on preliminary testing on RDTs. The dilution “L” was the last dilution with detactable RNA purified from RDT.

Zika virus (H/PF/2013, EVA 001v-EVA1545)– and CHIKV (H20235/STMARTIN/2013, EVA 001v-EVA1540)–inactivated strains provided by the European Virus Archive collection (https://www.european-virus-archive.com/) were 10-fold serial-diluted using Minimum Essential Media (MEM, Gibco, Thermo Fisher Scientific). Following RDT manufacturer instructions for NS1 testing, 100 µL of each virus dilution was loaded on the NS1 cassette of RDTs in triplicate and left for 2 hours for the specimen to dry. Each RDT was then opened, and a 15-mm strip piece was cut out from the sample pad, as described.^12^

For DENV1, each dilution was loaded onto 39 RDTs, three RDTs were immediately processed (D0), and the rest were divided into three groups and placed at three different temperatures: 4°C, −80°C, and 35°C. After different storage times, 2 days (D2), 1 week (W1), 1 month (M1), and 2 months (M2), three RDTs were taken out from each storage condition and then processed.

### RNA extraction.

As reported,^12^ each RDT strip fragment was incubated in 200 µL of ATL lysis buffer (Qiagen, Hilden, Germany) for 15 minutes at 56°C; 140 µL was then extracted using QIAamp Virus RNA Mini kit (Qiagen), following the manufacturer’s instruction (elution in 60 µL). As reference, 140 µL of each virus dilution, in triplicate, was also directly extracted contemporaneously, to be tested alongside the RDT extract.

### Real-time RT-PCR (RT-qPCR).

DENV, CHIKV, and ZIKV extracts were tested by the respective hydrolysis probe RT-qPCR assays (Table 1).^13–15^ SuperScript^™^ III Platinum^®^ One-Step qRT-PCR kit (Thermo Fisher Scientific) was used, following the manufacturer’s instruction, using 400 nM of each primer, 160 nM of probe, and 5 µL of extract in 25 µL reaction volume. Thermal cycling used was as follows: 15 minutes at 50°C, 2 minutes at 95°C, and 45 cycles (15 seconds at 95°C and 45 seconds at 60°C). For each tested virus, all samples were tested on the same plate.

**Table 1:** Sequences of the oligonucleotides used in this study for reverse transcription real-time polymerase chain reaction detection of dengue virus, chikungunya virus, and Zika virus RNA

Chikungunya: Pastorino et al.^14^	Forward primer	AAG CTY CGC GTC CTT TAC CAA G
	Probe	FAM-CCA ATG TCY TCM GCC TGG ACA CCT TT-TAMRA
	Reverse primer	CCA AAT TGT CCY GGT CTT CCT
Zika: Lanciotti et al.^13^	Forward primer	TTG GTC ATG ATA CTG CTG ATT GC
	Probe	FAM-CGG CAT ACA GCA TCA GGT GCA TAG GAG –TAMRA
	Reverse primer	CCT TCC ACA AAG TCC CTA TTG C
Dengue: Leparc-Goffart et al.^15^	Forward primer	AGG ACY AGA GGT TAG AGG AGA
	Probe	FAM-ACA GCA TAT TGA CGC TGG GAR AGA CC-TAMRA
	Reverse primer	CGY TCT GTG CCT GGA WTG AT

RNA extracted from neat strain was diluted in AVE buffer containing RNA carrier (10 ng/µL) (Qiagen), aliquoted, and stored at −80°C to be used as positive control. A negative control (no template) was included in each run.

The limit of detections (LODs), lowest virus dilution positive by RT-qPCR tested in triplicate, were compared for direct and RDT extractions, and between the different storage conditions for DENV.

## RESULTS

### Temperature stability.

DENV RT-qPCR results from the RDTs loaded with the three DENV1 dilutions and stored at different temperatures are displayed in Table 2 and supplemental data (Supplemental Table 1). DENV was successfully detected from the RDT stored at 35°C for 1 month for the three viral dilutions. For the “low-titer” dilution, DENV RNA could not be amplified for one of the three RDTs stored at 35°C for 1 week, and for the three RDTs stored at 35°C for 2 months. However, two of the three “low-titer” RDTs stored at 4°C were also found to be PCR negative after 1 month.

**Table 2:** Dengue RT-qPCR Cq for RNA purified from RDT stored at 4°C, −80°C, and 35°C at different time points for 2 months

	RT-qPCR results, mean Cq (SD)													
	RNA from direct extract*				RNA purified from RDT									
DENV1 dilution	H	M		L	H	M		L	H	M	L	H	M	L
Day 0	19.66 (0.31)	26.92 (0.48)		32.88 (0.22)	23.60 (0.42)	30.74 (0.44)		36.14 (0.51)						
RDT storage temperature	–				−80°C				+4°C			+35°C		
RDT storage duration														
2 days	19.81 (1.01)	26.44 (0.12)	32.65 (0.11)		23.18 (0.34)		30.54 (0.17)	36.46 (0.88)	23.77 (0.53)	30.12 (0.54)†	37.07 (0.76)	23.01 (0.25)	29.90 (0.34)	35.69 (0.03)
1 week	19.94 (0.10)	26.68 (0.35)	32.88 (0.25)		23.33 (0.47)		30.25 (0.61)	35.33 (0.56)	23.84 (0.14)	31.02 (0.15)	36.61 (0.35)	24.17 (0.51)	31.49 (0.35)	ND*
1 month	20.49 (0.60)	27.99 (0.36)	34.08 (0.48)		24.92 (0.43)		31.16 (0.16)†	38.70 (1.33)	26.05 (0.86)	33.06 (0.34)	ND‡	23.18 (0.17)	29.81 (0.28)	36.78 (0.66)
2 months	20.28 (0.32)	27.12 (0.01)	33.24 (0.22)		24.71 (0.07)		31.14 (0.33)	36.34 (0.08)	23.55 (0.17)	30.67 (1.09)	36.21 (0.46)	27.40 (1.11)	34.21 (0.42)	ND§

Cq = quantification cycle value; DENV = dengue virus; RDT = rapid diagnostic test; RT-qPCR = reverse transcription real-time polymerase chain reaction. RNA directly extracted, concomitant with RDT extraction, from aliquots of DENV1 isolate dilutions stored at −80°C, to serve as reference. DENV 1 dilutions correspond to 1/10 serial dilution, with H (high) corresponding to the dilutions with the highest titer and L (Low) to the lowest titer. M = medium. Mean of Cq is obtained for triplicate extractions. SD = standard deviation of Cq obtained for triplicate extractions. ND = no detection for at least one of the triplicate RDT (shaded in gray for better visibility).

* Reverse transcription real-time polymerase chain reaction was negative for 1/3 of triplicate RDTs.

† Result excluded for analysis because of technical issue.

‡ Reverse transcription real-time polymerase chain reaction was negative for 2/3 of triplicate RDTs.

§ Reverse transcription real-time polymerase chain reaction was negative for 3/3 of triplicate RDTs.

### Chikungunya virus detection.

CHIKV RT-qPCR data, for experiments performed on RNA directly extracted and extracted after loading on RDT for CHIKV dilutions 10^−4^–10^−9^, are presented in Table 3. The LOD for RNA extraction from RDT was 10^−7^, as compared with 10^−9^ for direct virus extraction. For all dilutions, standard deviation (SD) of quantification cycle value (Cq) from triplicate RDTs was ≤ 0.52; a similar result was observed for direct extraction with SD from 0.14 to 0.6 (excluding dilution at the LOD). This is indicative of good reproducibility of CHIKV RT-qPCR from RDTs.

**Table 3:** Chikungunya RT-qPCR results for direct vs. RDT RNA extract

Virus dilution	Direct RNA extract			RDT RNA extract		
	Cq	Mean Cq	SD of Cq	Cq	Mean Cq	SD of Cq
10^−4^	22.04	21.77	0.37	23.95	23.97	0.32
	21.35			24.29		
	21.93			23.66		
10^−5^	24.95	25.07	0.14	28.04	27.81	0.29
	25.03			27.48		
	25.23			27.91		
10^−6^	28.20	28.22	0.60	31.08	31.19	0.52
	27.64			31.76		
	28.83			30.74		
10^−7^	32.15	32.03	0.15	33.96	34.21	0.51
	32.08			34.79		
	31.86			33.87		
10^−8^	34.58	34.99	0.40	36.98	No Cq	–
	35.37			37.72		
	35.01			No Cq		
10^−9^	36.95	37.78	1.06	No Cq	No Cq	–
	38.97			No Cq		
	37.41			No Cq		

Cq = quantification cycle value; RDT = rapid diagnostic test; RT-qPCR = reverse transcription real-time polymerase chain reaction. All RT-qPCR results displayed in this table are from same PCR run. Limit of detection: lowest virus dilution positive by RT-qPCR tested in triplicate is shaded in gray.

### Zika virus detection.

ZIKV RT-qPCR data, for experiments performed on RNA directly extracted and extracted after loading on RDT for ZIKV dilutions 10^−2^–10^−8^, are presented in Table 4. The LOD for RNA extraction from RDT was 10^−6^, as compared with 10^−7^ for direct virus extraction. For all dilutions (except for the one at the LOD), standard deviation (SD) of Cq from triplicate RDTs was ≤ 0.37; a similar result was observed for direct extraction with SD from 0.13 to 0.27 (excluding dilution at the LOD). This is indicative of good reproducibility of ZIKV RT-qPCR from RDTs.

**Table 4:** Zika RT-qPCR results for direct vs. RDT RNA extract

Virus dilution	Direct RNA extract			RDT RNA extract		
	Cq	Mean Cq	SD of Cq	Cq	Mean Cq	SD of Cq
10^−2^	20.40	20.63	0.25	25.50	25.30	0.20
	20.89			25.30		
	20.61			25.10		
10^−3^	24.15	24.35	0.27	28.68	29.04	0.36
	24.65			29.05		
	24.24			29.40		
10^−4^	27.71	27.68	0.24	31.59	31.41	0.16
	27.90			31.35		
	27.42			31.29		
10^−5^	31.50	31.36	0.21	33.55	33.83	0.37
	31.45			33.68		
	31.12			34.25		
10^−6^	34.62	34.76	0.13	37.44	37.44	0.47
	34.79			37.91		
	34.88			36.98		
10^−7^	38.43	39.98	2.44	No Ct	No Cq	–
	42.79			No Ct		
	38.71			38.99		
10^−8^	43.78	No Cq	–	44.01	No Cq	–
	39.77			No Ct		
	No Ct			No Ct		

Cq = quantification cycle value; RDT = rapid diagnostic test; RT-qPCR = reverse transcription real-time polymerase chain reaction. All RT-qPCR results displayed in this table are from same PCR run. Limit of detection: lowest virus dilution positive by RT-qPCR tested in triplicate is shaded in gray.

## DISCUSSION

In the last decade, there has been major investment in much needed diagnostics for global infectious diseases.^16^ Although innovative technology is being introduced to strengthen health services in poor remote areas,^17^ success of these diagnostics depends on their accessibility and affordability in the areas that need them most.

In Laos, access to laboratory infectious disease diagnostics is largely confined to the capital city, Vientiane, and there is poor infrastructure for cold-chains to transport samples from provinces to the capital. Hence, minimal surveillance is currently possible for important public health threats such as DENV, CHIKV, and ZIKV.

As a development of a previous study,^12^ we show here that the storage of the used RDTs for a few weeks at elevated temperature does not compromise detection of DENV RNA by RT-qPCR. DENV RNA was purified and could be detected for the three tested dilutions even after 1 month storage of the RDTs at 35°C. Similar Cq were obtained in comparison to the RDTs tested at day 0. Some RNA degradation seems to occur after a 2-month storage at 35°C. For the dilutions “high” and “medium,” the RT-qPCR results were four Cq higher for the RDTs at 35°C than for the RDTs tested at day 0, and RT-qPCR was negative from the RDTs loaded with the “low” dilution. However, the “low” dilution was chosen to be at the LOD, and we showed in our previous study that the quantity of RNA recovered varies between RDTs.^12^ This may explain why DENV RT-qPCR was negative for some “low” RDTs tested after 1 month at 4°C, and 1 week and 2 months at 35°C.

This proof-of-principle study demonstrates the potential use of DENV RDTs for the storage, transport, and RT-qPCR co-detection of CHIKV and ZIKV as well as for DENV.^12^ CHIKV RNA was detected at 2 logfold lower dilution from RDT than direct extraction, and ZIKV RNA at 1 logfold lower dilution from RDT than direct extraction. As has been shown for DENV, less RNA is recovered when extracted from RDT as compared with direct viral extraction,^12^ 1–2 log differences, as observed in this study, would still enable detection of infected patients as shown for DENV with high agreement when comparing RT-qPCR results after direct and RDT extractions when tested with a panel of patient samples stored on RDTs.^12^ However, the practical utility of this system for enhancing surveillance in rural Asia requires testing of the RDTs using patient samples collected in the provinces, especially for ZIKV which is known to produce low viremia.^18^ It would need parallel development of guidelines for the inclusion criteria for performing the RDTs, enhancement of infrastructure for RDT shipping to a centralized laboratory for PCR assays, dashboard systems for the reporting of data to provincial hospitals and the Ministry of Health. In addition, funding of the dengue RDTs would be required to expand the coverage area, which is often limited to provincial hospitals and not yet available in remoter areas.

Our findings suggest that the distribution of SD BIOLINE Dengue DUO RDTs in rural areas could be extremely useful for the combined surveillance of dengue, Zika, and chikungunya viruses in Laos, and locations with similar environments. Although patients with the lowest viral titers would probably not be detected, especially for Zika infection, the detection of the patients with medium and high titers would permit the identification of epidemics. To date, in Laos, reports of ZIKV and CHIKV infections have been limited, despite a favorable climate for the rapid breeding of the vector and transmission of the viruses. There have been only 18 confirmed ZIKV cases detected from a retrospective analysis of sera, 2012–2013, and one ZIKV case was reported in 2016.^8^ Co-circulation of CHIKV during a DENV epidemic in 2013 in southern Laos has been reported.^19^ It is likely that CHIKV and ZIKV circulation have been underestimated and misdiagnosed as other febrile illnesses circulating in the areas in the absence of confirmatory diagnostic tests.

Rapid diagnostic tests have the potential to be a unique, simple, and cost-effective tool to improve laboratory detection of DENV, CHIKV, and ZIKV across wide geographical areas. Large-scale implementation of surveillance for these viruses is of crucial public health importance, to inform intervention strategies and for the early detection of epidemics. Combined surveillance is of particular importance in the current context of recent expansion of arboviral diseases because of increasing urbanization and expansion of long distance transportation.^20^

Further studies will be needed to evaluate this method under field conditions, including the practical aspects of patient sample collection in remote areas. The ability to harness novel technology to suit the setting has wider implications, to be transferred to other users and other diseases.

## Supplemental table

Supplemental materials
