# Supplementary material for: Harnessing Dengue Rapid Diagnostic Tests for the Combined Surveillance of Dengue, Zika, and Chikungunya Viruses in Laos
Source: Am J Trop Med Hyg. 2020 Mar 9;102(6):1244–8. doi: 10.4269/ajtmh.19-0881 (PMC7253103; doi:10.4269/ajtmh.19-0881)
Supplement: Supplementary file 1 [file tpmd190881.SD1.pdf]

Supplemental Table S1. Dengue RT-qPCR results for RNA purified from RDT stored at 4°C, -80°C and 35°C for two months.

DENV1 dilutions	RNA extract	Day 0				Day 2			Week 1			Month 1			Month 2		
		Cq value	M Cq	SD Cq		Cq value	M Cq	SD Cq	Cq value	M Cq	SD Cq	Cq value	M Cq	SD Cq	Cq value	M Cq	SD Cq
High	Ref1	19.97				19.24			20.03			21.18			19.97		
	Ref2	19.66	19.66	0.31		19.22	19.81	1.01	19.83	19.94	0.10	20.18	20.49	0.60	20.60	20.28	0.32
	Ref3	19.36				20.98			19.95			20.10			20.26		
Medium	Ref1	27.43				26.30			26.71			28.33			27.04		
	Ref2	26.84	26.92	0.48		26.53	26.44	0.12	27.01	26.68	0.35	28.01	27.99	0.36	27.12	27.12	0.01
	Ref3	26.49				26.47			26.32			27.62			27.19		
Low	Ref1	32.93				32.58			33.14			34.47			33.41		
	Ref2	33.08	32.88	0.22		32.61	32.65	0.11	32.64	32.88	0.25	33.55	34.08	0.48	33.32	33.24	0.22
	Ref3	32.64				32.77			32.85			34.21			32.99		
High	RDT1	24.04			Storage at -80°C	23.08			23.83			24.83			24.75		
	RDT2	23.54	23.60	0.42		23.56	23.18	0.34	23.25	23.33	0.47	25.39	24.92	0.43	24.62	24.71	0.07
	RDT3	23.21				22.91			22.90			24.54			24.74		
Medium	RDT1	31.12				30.36			29.88			31.27	31.16	0,16	31.52		
	RDT2	30.57	30.74	0.44		30.70	30.54	0.17	29.92	30.25	0.61	31.04			30.98	31.14	0.33
	RDT3	30.53				30.54			30.95			No Cq*			30.91		
Low	RDT1	35.85				36.79			35.97			37.29			36.43		
	RDT2	36.73	36.14	0.51		35.46	36.46	0.88	35.08	35.33	0.56	39.94	38.70	1.33	36.31	36.34	0.08
	RDT3	35.84				37.12			34.95			38.89			36.27		
High	RDT1				Storage at 4°C	23.19			23.97			25.65			23.36		
	RDT2					23.89	23.77	0.53	23.70	23.84	0.14	27.03	26.05	0.86	23.64	23.55	0.17
	RDT3					24.23			23.84			25.46			23.65		
Medium	RDT1					30.50	30.12	0.54	31.00			33.45			30.00		
	RDT2					29.73			30.88	31.02	0.15	32.87	33.06	0.34	31.92	30.67	1.09
	RDT3					24.68*			31.18			32.87			30.08		
Low	RDT1					36.19			36.99			39.78			36.74		
	RDT2					37.42	37.07	0.76	36.53	36.61	0.35	No Cq	NA	NA	36.00	36.21	0.46
	RDT3					37.58			36.32			No Cq			35.90		
High	RDT1				Storage at 35°C	22.74			23.59			23.18			28.62		
	RDT2					23.23	23.01	0.25	24.46	24.17	0.51	23.00	23.18	0.17	27.11	27.40	1.11
	RDT3					23.06			24.48			23.35			26.46		
Medium	RDT1					29.56			31.09			29.59			34.67		
	RDT2					29.92	29.90	0.34	31.72	31.49	0.35	29.70	29.81	0.28	33.85	34.21	0.42
	RDT3					30.23			31.66			30.12			34.13		
Low	RDT1					35.72			36.51	36.43	0.12	37.50			No Cq		
	RDT2					35.66	35.69	0.03	36.34			36.21	36.78	0.66	No Cq	NA	NA
	RDT3					35.70			No Cq	NA	NA	36.63			No Cq		

Ref = RNA directly extracted, concomitantly to RDT extraction, from aliquots of DENV1 isolate dilutions stored at -80°C, to serve as reference.
* result excluded for analysis because of technical issue. DENV1 dilutions correspond to 1/10 serial dilution with “High” corresponding to the dilutions with the highest titer and “Low” to the lowest titer. M Cq= mean of Cq values obtained for triplicate extractions. SD Cq= standard deviation of Cq values obtained for triplicate extractions.
